# A novel oral vaccine delivery system for enhancing stability and immune protection: bacterium-like particle with functional coating

**DOI:** 10.3389/fmicb.2024.1481514

**Published:** 2024-10-30

**Authors:** Xinqi De, Mingchun Gao, Zheng Jia, Hongkun Ren, Runhang Liu, Xinyao Zhou, Junjie Guo, Jiaqing Wang, Qi Yu, Nanzhu Qu, Fang Wang, Junwei Ge

**Affiliations:** ^1^Heilongjiang Provincial Key Laboratory of Zoonosis, College of Veterinary Medicine, Northeast Agricultural University, Harbin, China; ^2^National Key Laboratory for Animal Disease Control and Prevention, Harbin Veterinary Research Institute, Chinese Academy of Agricultural Sciences, Harbin, China

**Keywords:** oral delivery, subunit vaccines, bacterium-like particles, lipid membrane, antigen absorption

## Abstract

Bacterium-like particles (BLPs) have gained significant attention in vaccine development due to their potential as effective immune enhancers and antigen delivery systems. BLPs are generated by boiling lactic acid bacteria in an acidic solution and are devoid of proteins and nucleic acids, offering advantages in terms of ease of preparation, high safety, and good stability. Furthermore, by employing protein anchor (PA), heterogeneous antigens can be efficiently displayed on the surface of BLPs, resulting in enhanced delivery effectiveness. Despite these benefits, most BLP-based vaccines are currently administered via injection or intranasal delivery, with oral delivery remaining limited. This limitation is primarily due to the harsh environment of the gastrointestinal tract, which degrades the antigens displayed on the surface of these particles. To enhance the efficacy of oral immunization with subunit vaccines, we developed a simple and rapid method for self-assembling a lipid membrane onto the surface of BLPs vaccines, achieving an encapsulation efficiency of up to 99%, and the combination has good biosafety. The novel oral delivery system not only preserves the adjuvant activity of BLPs but also efficiently protects antigens from adverse gastrointestinal environments, increasing the absorption of the vaccine in intestinal Peyer’s patches (PPs). Oral immunization was required only once, and protection after the challenge was up to 100%. Furthermore, we observed rapid immunity and cross-protection. Transcriptome analysis of the small intestine suggested that immune enhancement probably be exerted by promoting the absorption and transport of antigens. Therefore, we posit that the design of this new oral delivery system presents a novel approach to advancing the development of oral subunit vaccines.

## 1 Introduction

Modern subunit vaccines are designed to contain highly recombinant and purified protein or peptide antigens in order to effectively induce protective immunity. They have enhanced purity, safety, stability, and manufacturing scalability (Van Herck et al., 2021). Subunit vaccines can avoid the safety concerns associated with inactivated or live-attenuated vaccines and promote immunity against rapidly mutant pathogens (Hou et al., 2023). However, subunit vaccines are less immunogenic and often require multiple immunizations, leading to increased overall production costs (Chen et al., 2023). A novel Lactic acid bacteria (LAB) surface display technology based on bacterium-like particles (BLPs), also termed gram-positive enhancer matrix (GEM), was first proposed and developed by Bosma et al. (2006). BLPs are the peptidoglycan (PG) skeleton left after thermal acid treatment of LAB, which retains the shape and size of LAB without any active cellular components, providing a high degree of safety and stability (Heine et al., 2015). BLPs primarily activate the innate immune system through Toll-like receptor (TLR) 2 and have been extensively researched as a promising adjuvant for vaccines targeting a variety of infectious diseases (Raya Tonetti et al., 2020). Furthermore, BLPs provide a suitable surface capable of presenting diverse heterologous proteins via a peptidoglycan-binding domain, referred to as the protein anchor (PA) (Su et al., 2023). The innovative antigen delivery system enhances the efficiency of antigen delivery and enables the simultaneous presentation of multiple antigens on the particle surface. It facilitates the concurrent delivery of adjuvants and multiple antigens to antigen-presenting cells (APCs), thereby triggering an effective immune response (Tang et al., 2019; Seyfoori et al., 2021). To date, BLPs have been utilized in over 40 distinct vaccine designs targeting bacterial, viral, and parasitic pathogens. These vaccines have demonstrated 100% protection against influenza virus, Newcastle disease virus, *Streptococcus pneumoniae*, and *Plasmodium berghei* (Zhou et al., 2023).

Although BLPs largely compensate for the limited immunogenicity of subunit vaccines, much research on BLP-based vaccines primarily focuses on intranasal or injectable administration, with fewer studies exploring oral administration (Zhou et al., 2023). It is well known that traditional intramuscular vaccines face several challenges, including the difficulty of establishing an immunoprotective barrier at mucous membranes, and the risk of allergic reactions and infections (Ye et al., 2023). Intranasal administration enhances mucosal immunity but is characterized by inconvenience and time consumption. Conversely, oral administration is currently considered the gold standard for therapeutic drug delivery due to its convenience and non-invasive nature. Oral vaccination elicits systemic as well as mucosal immune responses, conferring an extra immunological advantage by protecting against infections of the mucosal surface (Lycke, 2012). Despite the widespread agreement on the desirability of oral vaccination, very few oral vaccines have received clinical approval (Coffey et al., 2021). It has been shown that the ability of oral administration of BLP-based vaccines to activate the immune system is not as good as that of intranasal administration (Audouy et al., 2006). This is primarily attributed to the numerous challenges the gastrointestinal tract poses for subunit vaccine delivery, encompassing the disintegration of biological agents and the restriction of mucosal barriers to their absorption (Rupassara et al., 2022). Additionally, in our previous study, new BLPs were innovated by thermal acid treatment of various Lactobacillus strains in the pre-laboratory stage, and BLP23017 was selected from them, which proved to be safe and efficient (Guo et al., 2024). A BLP23017-based multi-epitope vaccine was designed that proved to be immunopotentiated, but its immune efficacy by oral delivery also remains unsatisfactory.

Several strategies have been used to enhance the bioavailability of orally delivered vaccines, with encapsulation being a commonly employed and effective method. Encapsulation has been employed to safeguard vaccines from degradation by acids and enzymes in the gastrointestinal tract (Anselmo et al., 2016), utilizing materials such as chitosan, yeast microcapsules, hydrogel microcapsules, and bacterial outer membrane vesicles to enhance vaccine stability in the gastrointestinal environment. Moreover, chelating agents, organic solvents, lipids, bile extracts, and polymers have been thoroughly investigated to improve the oral bioavailability of vaccines. These compounds function through either the transcellular or paracellular route, disrupting the intestinal epithelium to facilitate vaccine absorption (Aungst, 2012). Despite their potential for oral administration, these methods have shown limited effectiveness and low survival efficiency in the complex gastrointestinal environment. Additionally, the preparation procedures are numerous and the separation process is cumbersome, impeding scalable production. Consequently, new approaches are necessary to address these limitations in oral delivery, paving the way for the oral administration of subunit vaccines.

**Figure 1:**
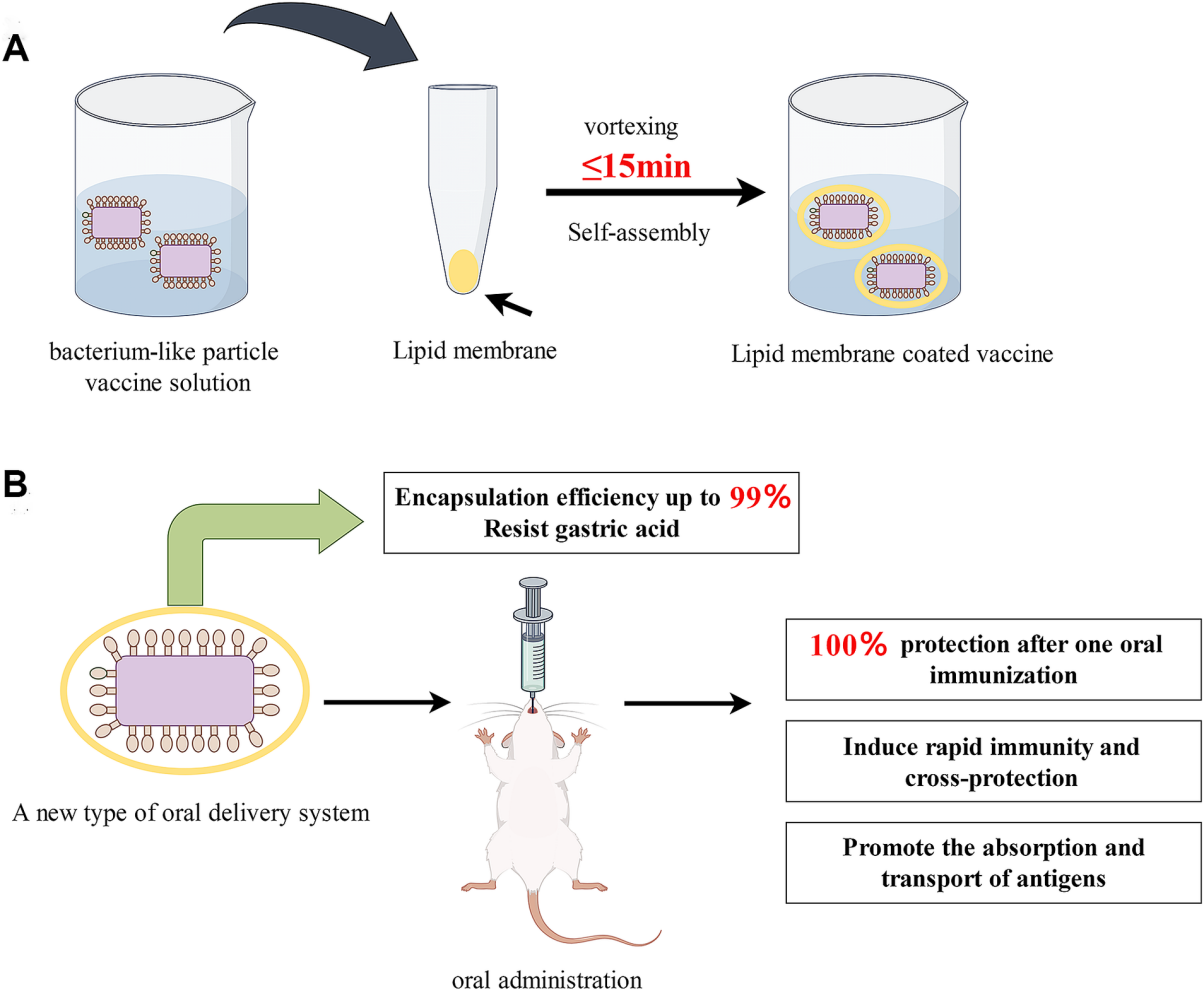
**(A)** Schematic illustration of the preparation of lipid membrane-coated bacterium-like particle vaccine; **(B)** Advantages of prepared LM@COB17. By Figdraw.

This study aimed to enhance the inadequate oral immunization efficacy of subunit vaccines. We developed a novel oral delivery system that is a display system for BLPs covered by lipid membranes and then assessed its potential immune-enhancing effect following oral immunization. For this purpose, (i) A rapid and efficient method was utilized to encapsulate the BLPs vaccine (named COB17, prepared in our lab) within lipid membranes in just 15 min, resulting in the formation of the encapsulated vaccine LM@COB17 (Figure 1); (ii) Transmission electron microscopy, flow cytometry, and fourier transform infrared spectroscopy were used to confirm the success and efficiency of the encapsulation; (iii) Transmission electron microscopy and flow cytometry were utilized to verify the protective impact of lipid membrane on the antigen following simulated gastroenteric fluid exposure; (iv) Fluorescence microscopic observation of frozen sections of intestinal Peyer’s patches (PPs) to verify the absorption of the vaccine; (v) The effect of LM@COB17 on oral immunity was evaluated through examining the protective impact upon challenge, humoral immunity, mucosal immunity, and other relevant indicators in a mouse model; (vi) Analyzing the rapid immune protection and cross-protection in mice; (vii) The mechanism through which LM@COB17 exerts immune-enhancing effects was analyzed using transcriptome analysis. Our results demonstrated that LM@COB17 provided effective protection to the antigen in the adverse gastrointestinal environment and enhanced its absorption in intestinal PPs, resulting in an improved oral immune effect of the vaccine, achieving 100% protection after a single oral immunization.

## 2 Materials and methods

### 2.1 Materials

DOPA [1,2-dioleoyl-sn-glycero-3-phosphate (sodium salt)], cholesterol, and Chloroform were purchased from Aladdin (Shanghai, China). The model vaccine, COB17, is a multi-epitope vaccine displayed by a BLP vector and demonstrates favorable biocompatibility, and strong immunoprotection against *Clostridium perfringens* (*C. perfringens*) (Niu et al., 2024). *C. perfringens* type A (C57-8) was purchased from the China Veterinary Drug Administration and the IPEC-J2 (small intestinal epithelial cells of pigs) Professor Zhanyong Wei of Henan Agricultural University gifted cell. *Levilactobacillus brevis* 23,017 (*L. brevis* 23,017) strain was maintained in our laboratory.

### 2.2 Preparation of BLP-based *Clostridium perfringens* multi-epitope vaccine

The vaccine was prepared according to the method established in our laboratory. Briefly, *L. brevis* 23,017 from 100 mL of freshly prepared overnight cultures was harvested and rinsed with sterile distilled water. The bacterial precipitate was then suspended in 20 mL of 10% trichloroacetic acid and boiled for 45 min. Following this, the precipitate underwent three washes with 20 mL of sterile phosphate-buffered saline (PBS) and was ultimately resuspended in 10 mL of PBS, and named BLP23017 (Guo et al., 2024). CPMEA is a multi-epitope antigen from *C. perfringens*. The model antigen CPMEA was bound to the surface of BLP23017 through the anchoring protein OACD According to our previous research, the final product is CPMEA-OACD-BLP23017, abbreviated as COB17 (Niu et al., 2024).

### 2.3 Preparation of COB17 coated with lipid membrane

The wrapping of lipid membranes was performed by previous researchers recently (Cao et al., 2019). In short, COB17 containing 300 micrograms of antigen was washed and resuspended in 1 mL of calcium phosphate solution with 12.5 mM of CaCl_2_. DOPA and cholesterol were dissolved in 8 mL of chloroform at a 4:1 molar ratio. A rotating evaporator (INDUCTION MOTOR, China) was used to dry the solution at room temperature, leading to the formation of lipid membranes. The lipid membranes were hydrated in 1 mL of COB17 solution and vortexed (Kirin Medical Instrument Factory, China) for 15 min. Finally, the resultant solution was stored at −80°C for further characterization. The prepared lipid membrane encapsulated COB17 was named LM@COB17.

### 2.4 Characterization of LM@COB17

The morphology of LM@COB17 and COB17 were characterized utilizing transmission electron microscopy (TEM) (Hitachi, Japan). Dynamic light scattering (DLS) (Malvern Panaco Instruments, United Kingdom) measurements were used to detect the average size and zeta potential of LM@COB17 and COB17. Combining fourier transform infrared (FTIR) spectroscopy (Thermo Fisher Scientific, Germany), LM@COB17 and COB17 were characterized to gain insight into their surface elemental composition. COB17 coated with lipid membranes labeled by FITC was examined by flow cytometry (Becton, Dickinson and Company, USA), to detect the encapsulation efficiency of lipid membranes.

### 2.5 *In vitro* cytotoxicity assays

The *in vitro* cytotoxicity of LM@COB17 was tested in IPEC-J2 (small intestinal epithelial cells of pigs) cell. Briefly, IPEC-J2 cells were seeded in a flat-bottom 96-well plate (Thermo Fisher Scientific, China) at 5000 cells/well, in a DMEM culture medium. Next, the cultures were incubated with 100 μL LM@COB17 solution in the different concentrations of 1 × 10^9^, 2 × 10^9^, 3 × 10^9^, and 4 × 10^9^ CFU/mL for 6 h or 24 h. Cells treated with DMEM and without LM@COB17 were taken as control wells. The wells with DMEM and LM@COB17 added (without cells) were blank wells. Then, 10 μL of CCK8 solution was added and incubated for another 30 min at 37°C. The OD450 value of the culture was recorded. Cell viability (%) = (sample-blank)/(control-blank) × 100%.

### 2.6 *In vivo* toxicity assessment

The female Kunming mice (4–6 weeks) were divided into two groups (six mice per group) and given PBS or 1 × 10^8^ CFU LM@COB17 orally on days 0, 2, 4, and 6. The weight of the mice was monitored daily. The mice were euthanized on day 10 via cervical dislocation as per the standard operating procedures approved by the Institutional Animal Ethics Committee, and their major organs were collected and submitted to Wuhan Sevier Biotechnology Co., Ltd. for histopathological evaluation using H&E staining.

### 2.7 Stability studies

LM@COB17 was stored at 4 ± 1°C and 27 ± 2°C, respectively. The difference in particle size and zeta potential was found by DLS (Malvern Panaco Instruments, United Kingdom) after a time interval of 30, 60, and 90 days.

### 2.8 Stability of LM@COB17 in simulated GI fluids

To demonstrate the protective effect of lipid membranes in the gastrointestinal (GI) tract environment, we utilized TEM imaging (Hitachi, Japan) to visualize the morphological changes in LM@COB17 and COB17 after exposure to simulated gastric fluid (SGF) and simulated intestinal fluid (SIF) for 3 h. Additionally, the stability and protective effect of the lipid membrane in SGF and SIF were assessed using flow cytometry (Becton, Dickinson, and Company, USA). On one hand, we utilized FITC to label the lipid membrane and subsequently observed its retention on the vaccine surface. On the other hand, we employed flow cytometry to determine the protective effect of the lipid membranes by quantifying the number of particles in the LM@COB17 and uncoated COB17 solutions. Specifically, the number of particles ungated every 10 s was calculated at a consistent flow rate. In short, LM@COB17 labeled with FITC, COB17, and LM@COB17 were resuspended into 1 mL of SGF and SIF, and incubated for 3 h at 37°C. Subsequently, they were cleaned three times with deionized water.

### 2.9 Absorption of COB17 or LM@COB17 in intestinal PPs after oral administration

The FITC-labeled COB17 and FITC-labeled LM@COB17 solutions were resuspended in PBS (1 × 10^9^ CFU/mL). The female Kunming mice (*n* = 5) were orally administered with 1 × 10^8^ CFU/mouse COB17-FITC or LM@COB17-FITC. 24 h after administration, the mice were euthanized via cervical dislocation. The resected PPs were fixed in 4% paraformaldehyde (PFA) at 4°C for 1 h, then transferred to a 20% sucrose solution for 1 h. The fixed tissues were then embedded in the OCT freezing compound provided by the manufacturer (Servicebio, China) and rapidly frozen using liquid N2. The frozen tissues were cut into 6 mm-thick slices, after which nuclear staining was performed (Solarbio, China), and the images were observed using a fluorescence microscope (Leica, China).

### 2.10 Experimental animals

The Liaoning Changsheng Biotechnology Co., Ltd. provided the female Kunming mice, which ranged in age from 4 to 6 weeks. The mice were housed under controlled environmental conditions (25 ± 1°C and 65% humidity) with access to food and water, following a 12-h light/dark cycle, three mice per cage. After acclimatizing to the laboratory environment for 1 week, the mice were randomly assigned to groups.

The Ethical Committee of the Institute approved all scientific experiments. All applicable international and national guidelines for the care and use of animals in experiments were followed and approved by the Institutional Committee of Northeast Agricultural University (NEAUEC20210326).

### 2.11 Animal groups, vaccination, and challenge

A total of 30 mice, 12 were randomly selected as the blank control and infection groups respectively, 6 in each group. The remaining 18 mice were randomly divided into 2 groups, the COB17 and LM@COB17 groups, with 9 mice in each group. Control and infection groups: 200 μL of PBS was orally administered; COB17 group: 200 μL of COB17 (including 50 μg of antigen) was orally administered; LM@COB17 group: 200 μL of LM@COB17 (including 50 μg of antigen) was orally administered.

On day 0, each mouse in COB17 and LM@COB17 groups (*n* = 9) was orally administered 200 μL, containing a dosage of 50 μg of antigen.

After 3 days of oral immunization, 3 mice from the COB17 and LM@COB17 groups, respectively, were randomly selected to be euthanized. The duodenum samples were sent to BGI Genomics Co., Ltd. (Shenzhen, China) for whole transcriptome sequencing.

Following a 21-day period, 2 × 10^8^ CFU of *C. perfringens* type A (C57-8) were injected intraperitoneally into the mice of the infection group, COB17 and LM@COB17 groups (*n* = 6). Over the course of the next 7 days, observations and records were made regarding changes in body weight, clinical symptoms, and survival rates for each group. All mice were euthanized via cervical dislocation 7 days after the challenge. Samples of sera, feces, spleen, ileum tissue, and jejunal intestinal mucus were taken for subsequent experiments.

On days 7, 14, 21, and 28 following vaccination, tail vein serum and fecal samples were taken in order to track the antibody response.

### 2.12 Antibody measurement

An indirect ELISA method was employed to assess the specific antibodies present in mice serum and feces. Approximately 3 mg/mL of purified CPMEA recombinant protein was utilized as the antigen for coating. Furthermore, peroxidase-conjugated goat anti-mouse IgG antibodies (A0216, Beyotime, Shanghai, China) and peroxidase-conjugated goat anti-mouse IgA antibodies (RS030211, Immunoway, Texas, United States) were employed. Additionally, the amounts of total intestinal mucus SIgA were ascertained using an ELISA kit obtained from Shanghai Shengong bio-engineering Co., Ltd.

### 2.13 SIgA-related cytokines measurement

TGF-*β* and IL-5 levels in serum samples on day 28 after immunization were assessed using commercially available ELISA kits (Boster Bioengineering Co., Ltd., Wuhan), according to the manufacturer’s instructions.

### 2.14 Real-time quantitative PCR

Real-time quantitative PCR (RT-qPCR) was utilized to assess the manifestation of IL-4, IL-10, IL-1*β*, and IFN-*γ* in spleen tissue samples on day 28 after immunization. Primers for this study were synthesized with the company (Comate Bioscience Co., Ltd., Changchun, Jilin, China) and provided in Supplementary Table S1 according to the reference (Pan et al., 2022; Shi et al., 2022). RT-qPCR kits were obtained from EnzyArtisan Technology Co., Ltd. (Shanghai) and employed following the established method. The PCR conditions comprised an initial denaturation for 30 s at 95°C, followed by 39 cycles of 10 s at 95°C, 15 s at 60°C, and 30 s at 72°C. The housekeeping gene β-actin was used for normalization, and the relative mRNA level was computed using the 2^−△△Ct^ technique. The experiment was conducted in three replicates.

### 2.15 Toxin neutralization test

The *in vitro* toxin neutralization test was established following the previously published methodology (Goossens et al., 2016). The supernatants of *C. perfringens* type A cultured in cooked meat medium were cultured on the blood plate at 37°C overnight, and it was observed that the internal completely hemolytic ring caused by *θ*-toxin and the external incomplete hemolytic ring caused by *α*-toxin appeared on the blood plate. The supernatant of *C. perfringens* type A was mixed with the serum of mice in the immune group in equal volume, and incubated at 37°C for 2 h. 10 μL of the mixed drops were placed on the blood plate, and the hemolysis was evaluated after overnight incubation. The experiment was conducted in three replicates.

### 2.16 Histopathological detection

The ileum tissue samples from both the control, infected, and treated mice were preserved in 10% paraformaldehyde at normal temperature. Pathological sections were prepared and analyzed by Wuhan Service Technology Co., Ltd. (Wuhan, China).

### 2.17 Rapid immunity and cross-protection

The mice were randomly divided into 4 groups for different experiments, 6 mice per group and 24 mice in total.

On days 2 and 3 after the oral administration of LM@COB17, the mice were intraperitoneally challenged with 2 × 10^8^ CFU of the *C. perfringens* type A.

On days 3 and 13 after the oral administration of LM@COB17, the mice were intraperitoneally challenged with *Listeria monocytogenes* (ATCC19111), *Enterococcus faecalis* (ATCC29212), *Staphylococcus aureus* (CMCC26003), *Streptococcus suis* (ATCC700794), and *Escherichia coli* (CVCC230) at a dose of 1×10^8^ CFU.

The survival rates of the mice were observed and recorded over the following 7 days.

### 2.18 Transcriptome sequencing data analysis

To ensure data integrity, we used MD5 verification software to verify the original data. Subsequently, gene functional annotation, differential expression analysis, KEGG enrichment analysis, and GO enrichment analysis were performed. The STRING database and Cytoscape were utilized to analyze and establish the protein–protein interaction (PPI) network. The differentially expressed genes (DEGs) were identified based on *p* < 0.05 and | log2 (fold change) | > 1.

### 2.19 Statistical analysis

The statistical analysis was conducted using GraphPad Prism 8.0.1 software. The experimental results are presented as the mean ± SD. The significance of group differences was assessed using one-way or two-way ANOVA. Statistical significance was denoted as *p < 0.05, **p < 0.01, ***p < 0.001.

## 3 Results

### 3.1 Preparation and characterization of LM@COB17

**Figure 2:**
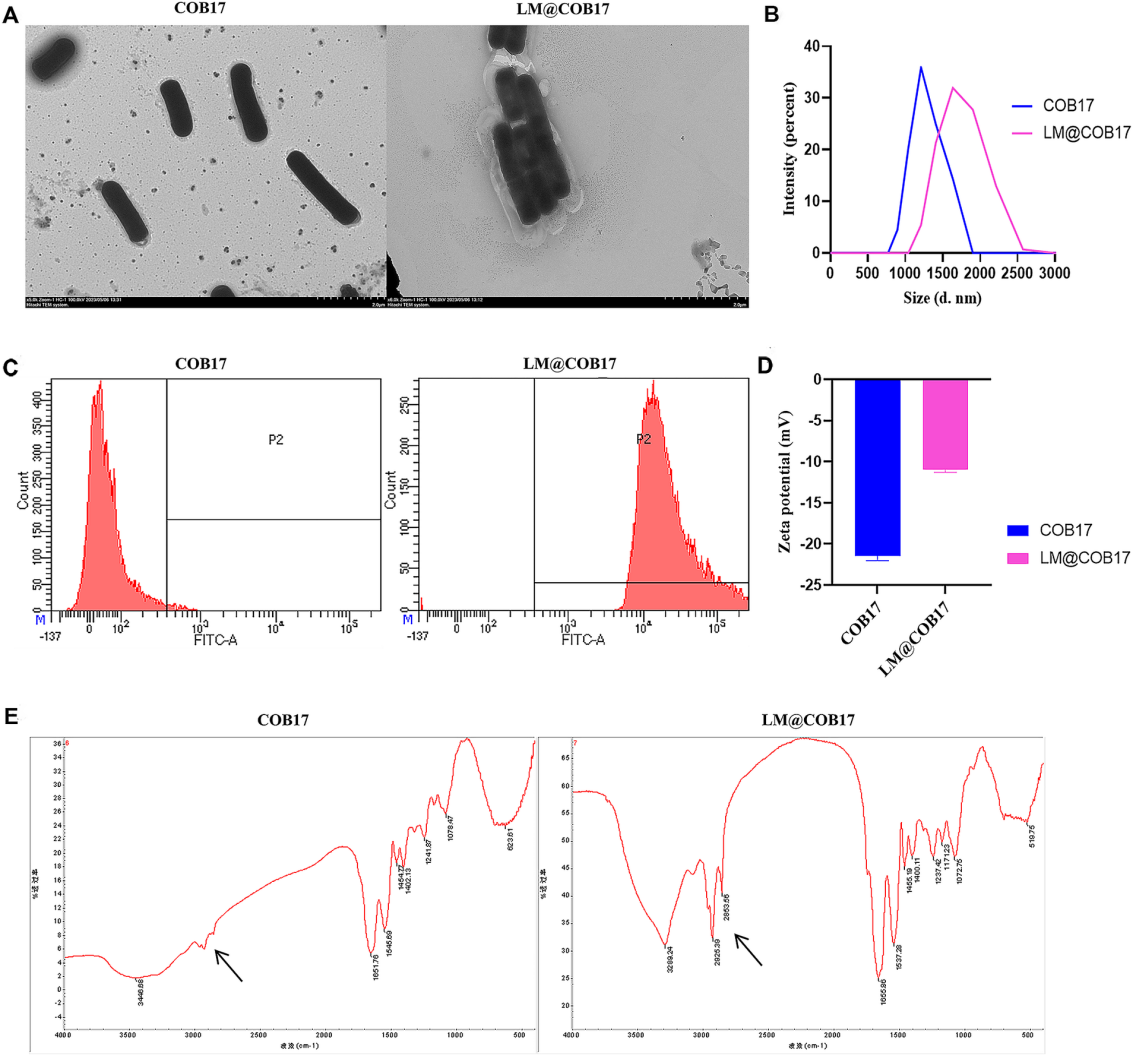
Characterization of LM@COB17. **(A)** TEM images of COB17 and LM@COB17. Scale bars, 2 μm; **(B)** Size distribution of COB17 and LM@COB17; **(C)** Flow cytometric analysis of FITC-labeled lipid membrane. COB17 was used as controls; **(D)** Zeta potential of COB17 and LM@COB17; **(E)** FTIR spectrum of COB17 and LM@COB17.

The BLPs vaccine produced in our laboratory was coated with lipid membranes using vortexing. TEM images depicted a distinct additional outer shell on the coated COB17, contrasting with the sharp edge of the uncoated COB17, providing further evidence of successful encapsulation within the lipid membranes (Figure 2A). DLS measurements disclosed the size of COB17 increased from 1,039 to 1,635 nm, and the zeta potential increased from −21.44 to −10.96 mV (Figures 2B,D), showing both the size and zeta potential of COB17 increased after lipid membrane coating.

Through FTIR analysis, we observed that LM@COB17 exhibits different characteristic peaks compared to COB17 (Figure 2E), this indicated that LM@COB17 contained both components of COB17 and lipid membranes. Flow cytometry measurements of COB17 coated with FITC-labeled lipid membranes showed a significant increase in fluorescent intensity compared to uncoated COB17, indicating the presence of the coating membranes on the surface of COB17. Furthermore, an encapsulation efficiency of up to 99% was achieved (Figure 2C).

### 3.2 LM@COB17 had strong biocompatibility

**Figure 3:**
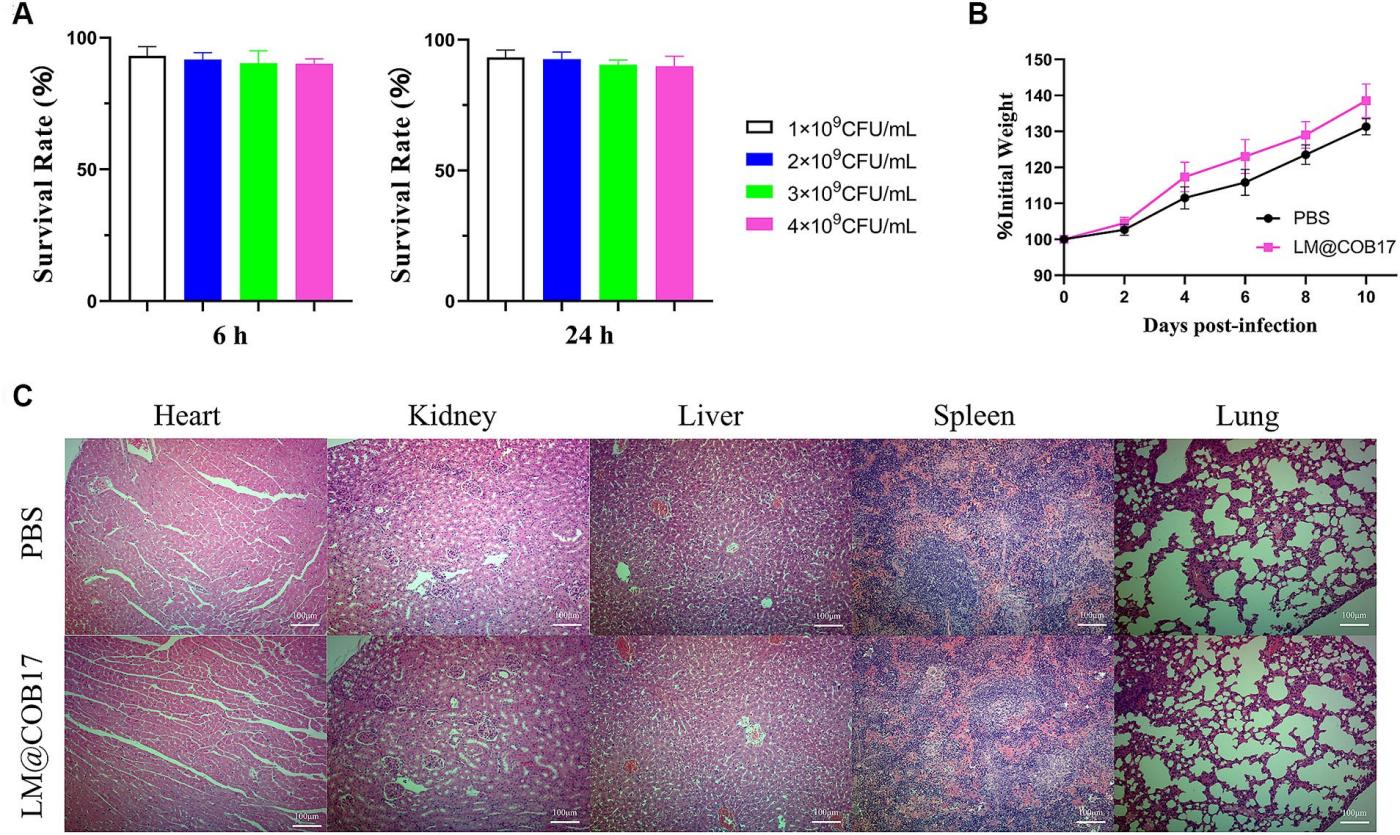
The biocompatibility of LM@COB17. **(A)** Cell survival rates (%) of IPEC-J2 when exposed to LM@COB17. **(B)** The body weight of mice treated with PBS or LM@COB17 on days 0, 2, 4, and 6 through gavage administration. **(C)** Representative H&E staining images of the major organs of mice treated with PBS or LM@COB17. Scale bar: 100 μm (6 mice per group).

The cytotoxicity of LM@COB17 was assessed using the CCK8 assay on IPEC-J2 cells. As demonstrated in Figure 3A, the cell viability of IPEC-J2 cells was over 90% after incubated with LM@COB17 solution in the different concentrations of 1 × 10^9^, 2 × 10^9^, 3 × 10^9^, and 4 × 10^9^ CFU/mL for 6 h or 24 h, suggesting the negligible cytotoxic effects of LM@COB17.

Furthermore, there were minimal differences in the body weights and histological sections of major organs between the LM@COB17-treated mice and the healthy mice (Figures 3B,C). All of these results indicate that LM@COB17 did not cause any adverse side effects.

### 3.3 LM@COB17 was stable at 4°C and room temperature

The stability of LM@COB17 and COB17 over time was examined at 4 ± 1°C and 27 ± 2°C, and the particle size and zeta potential were measured at specific time points using DLS analysis, as presented in Table 1. No significant changes in the zeta potential of LM@COB17 were observed up to 30, 60, and 90 days at 4 ± 1°C and 27 ± 2°C. A slight increase in particle size was noted, likely due to aggregation over time. This suggests that LM@COB17 remained stable during this period.

**Table 1:** Effect of storage time on the particle size (nm) and Zeta potential (mV) of prepared LM@COB17 at different temperatures.

Temperature (C)		Storage time (Days)			
		Initial	30	60	90
4 ± 1	size (nm)	1,635 ± 4.32	1,647 ± 5.10	1,677 ± 4.08	1,690 ± 2.94
27 ± 2	size (nm)	1,635 ± 4.32	1,669 ± 4.97	1,693 ± 6.38	1704 ± 4.32
4 ± 1	Zeta potential (mV)	−10.96 ± 0.27	−12.55 ± 0.46	−13.03 ± 0.13	−13.23 ± 0.06
27 ± 2	Zeta potential (mV)	−10.96 ± 0.27	−13.22 ± 0.08	−13.25 ± 0.04	−13.78 ± 0.12

Data are represented as mean ± SD (*n* = 3).

### 3.4 The stability of LM@COB17 in the simulated gastroenteric fluid was significantly improved

**Figure 4:**
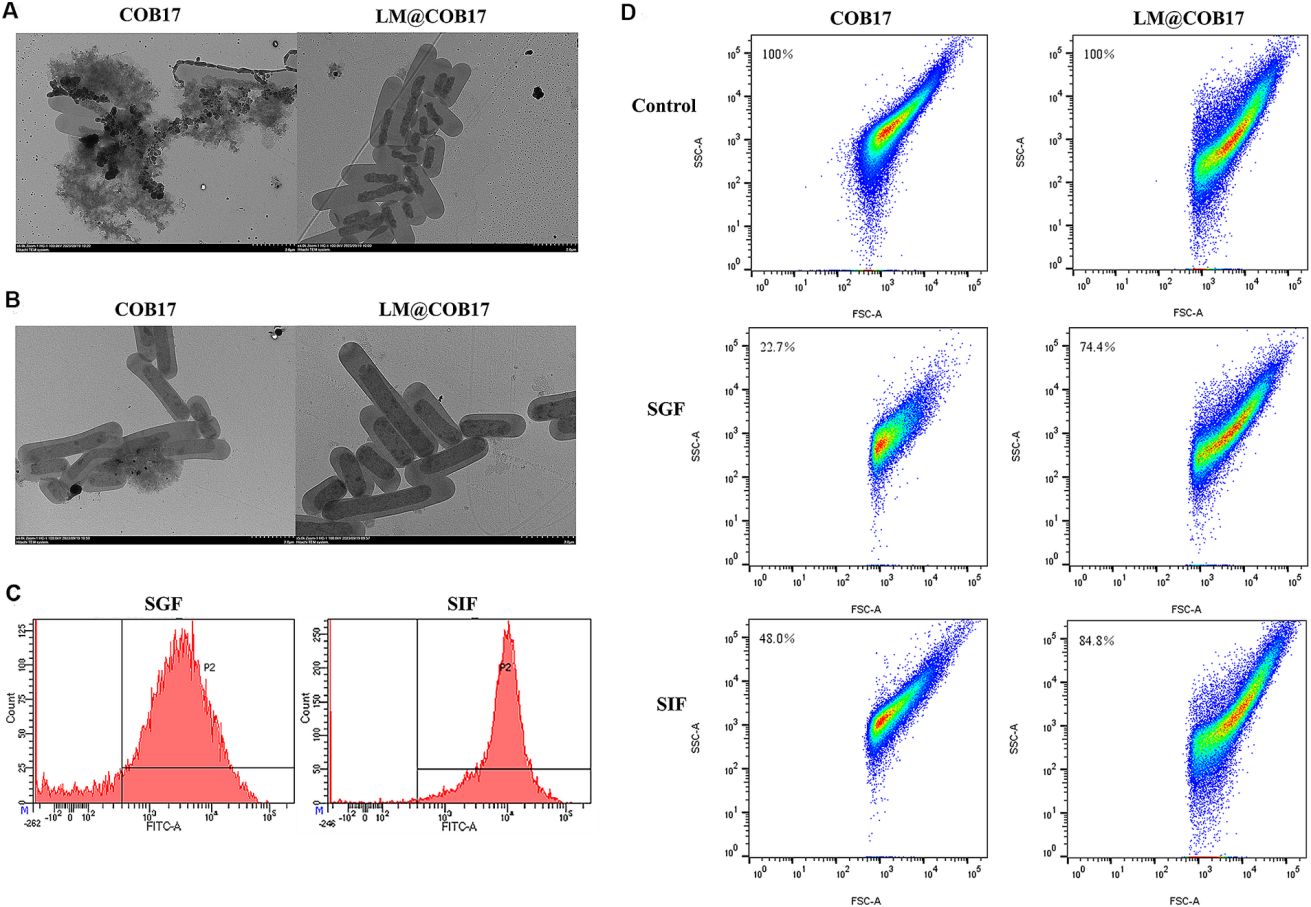
Stability of LM@COB17 in simulated GI fluids. **(A,B)** Typical TEM images of COB17and LM@COB17 cultured in SGF or SIF at 37°C for 3 h. Scale bar: 2 μm. **(C)** FITC-labeled lipid membrane were assessed by flow cytometry following 37°C incubation in SGF or SIF. **(D)** The number of remaining particles in the COB17 and LM@COB17 solution was detected by flow cytometry following 37°C incubation in SGF or SIF.

TEM imaging was used to investigate the stability of lipid membranes in simulated gastrointestinal fluid and to explore the morphological changes in COB17 following exposure to digestive tract solutions. The results, presented in Figures 4A,B, indicated that after 3 h of incubation in SGF, the external structure of the unpacked COB17 was damaged, leading to the outflow of its contents. In contrast, LM@COB17 showed less damage, with the contents severely shriveled. TEM images following 3 h of incubation in SIF revealed relatively mild structural damage to both COB17 and LM@COB17.

Flow cytometry results demonstrated that only about 20% of the lipid membrane was shed after 3 h of treatment in the SGF, demonstrating the relative stability of the coating membranes in SGF and their retention during gastric emptying. In the SIF for 3 h, less than 10% of the lipid membrane was shed (Figure 4C).

Additionally, the particle number of the vaccine in the LM@COB17 and unwrapped COB17 solution was quantitatively detected after treatment with simulated gastroenteric fluid by flow cytometry. It was observed that the particle number of the vaccine was less consumed in the LM@COB17 solution (Figure 4D). Following 3 h of treatment in SGF, the number of vaccine particles in solution COB17 decreased significantly, by approximately 77%, whereas the number of vaccine particles in solution LM@COB17 decreased by only about 26%.

### 3.5 LM@COB17 were absorbed more than COB17 in PPs

**Figure 5:**
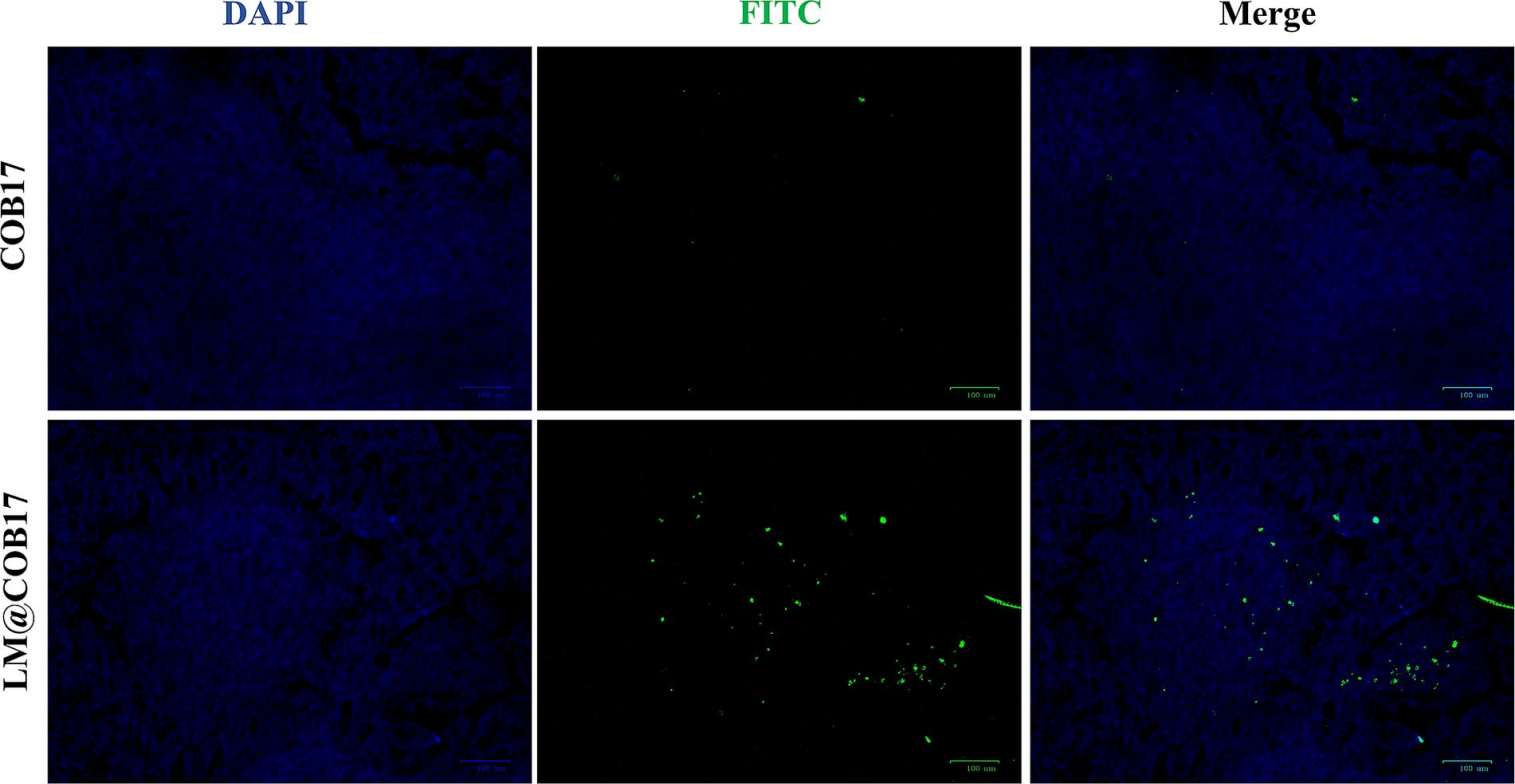
The absorption of COB17 and LM@COB17 in the PPs. Scale bar: 100 μm.

To elucidate the uptake of orally administered LM@COB17 and COB17 into the PPs, we analyzed frozen sections of mouse PPs after 24 h of oral administration of COB17-FITC and LM@COB17-FITC by fluorescence microscopy (Figure 5). Compared with the COB17 group, there was more green fluorescence in the PPs of the LM@COB17 group, indicating that more was taken up. Thus, the results suggest that LM@COB17 as an antigen delivery platform can target PPs more effectively and increase their uptake by PPs.

### 3.6 LM@COB17 improved the oral immunity effect of the vaccine

**Figure 6:**
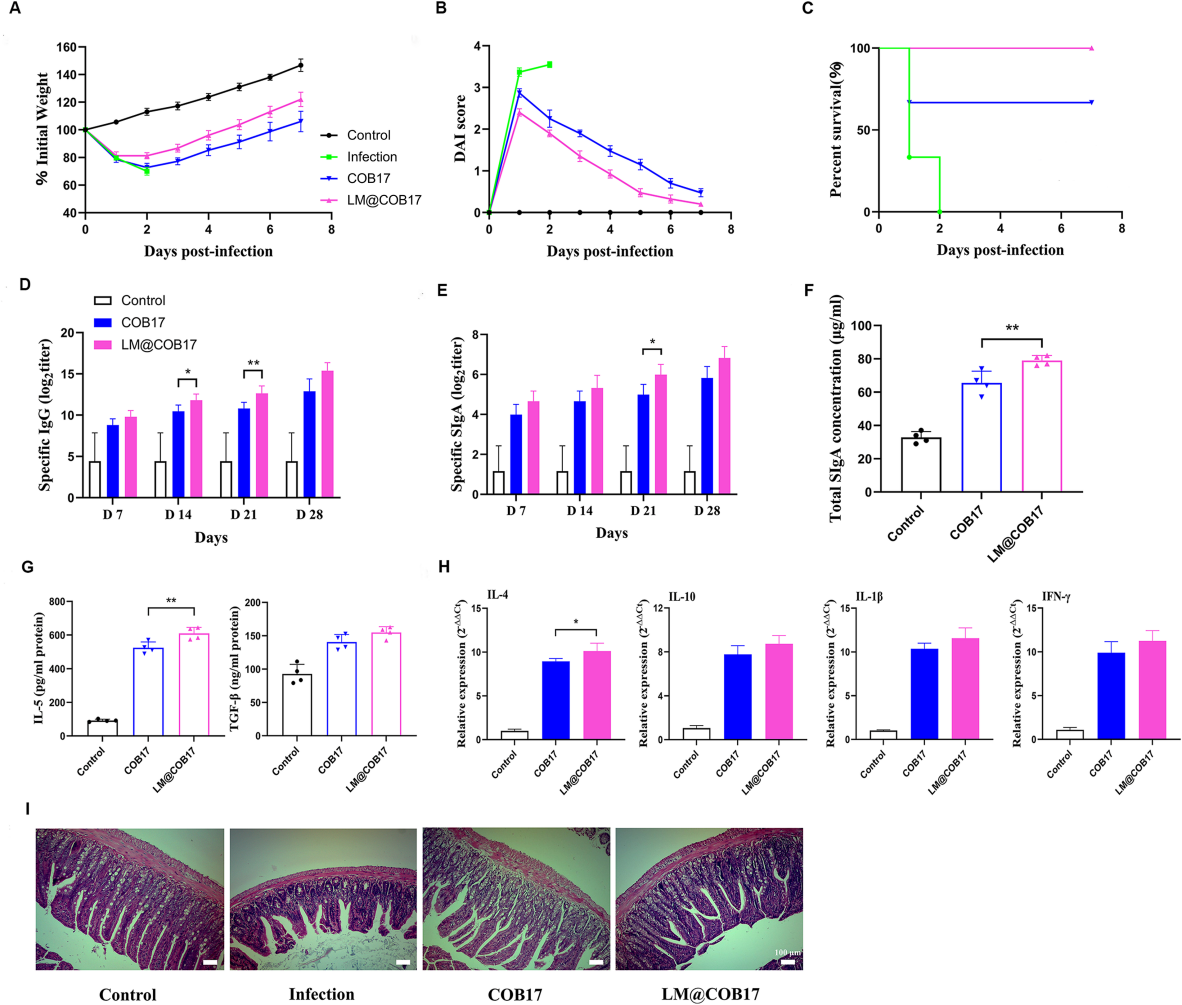
Evaluation of immune enhancement effect of LM@COB17. **(A)** Changes in body weight of mice after challenge. **(B)** DAI score of mice after challenge. **(C)** Survival rate of mice after challenge. **(D,E)** The titers of specific IgG antibodies in sera and specific SIgA antibodies in feces were measured on days 7, 14, 21, and 28 after immunization. **(F)** The concentration of total SIgA in intestinal mucus. **(G)** Results of cytokines related to SIgA in sera on the 28th day following immunization. **(H)** Results of immune-related cytokines in spleen cells on the 28th day following immunization. **(I)** The ileum tissue samples were collected on day 28 following immunization to detect pathological alterations (magnification of ×40). (Before challenge, *n* = 6; After challenge, two mice in COB17 group died, *n* = 4. *p ≤ 0.05; **p ≤ 0.01).

Our findings that LM@COB17 had good stability in simulated gastroenteric fluid encouraged us to further evaluate whether it enhanced the oral immunity effect of vaccines. As displayed in Figures 6A–C, mice in the LM@COB17 group exhibited superior acceleration in weight recovery contrasted with the COB17 group. The DAI score of mice in the LM@COB17 group was lower than the COB17 group. Furthermore, the survival rate of mice immunized with COB17 was 70%, whereas the survival rate of those in the LM@COB17 group was 100%. Consequently, LM@COB17 exhibited better protective effects.

To evaluate the antibody levels in the mice, we measured the levels of antigen-specific IgG antibodies in the serum and IgA antibodies in the fecal dilutions at 7, 14, 21, and 28 days post-immunization. As depicted in Figure 6D, on the 7, 14, 21, and 28 days following vaccination, the IgG content in the control group remained constant, while it steadily increased in the COB17 and LM@COB17, with the LM@COB17 group exhibiting the highest levels. This indicated that LM@COB17 significantly outperformed COB17 in enhancing antigen-specific humoral immunity. Similarly, we found the IgA levels in the COB17 and LM@COB17 groups were elevated (Figure 6E), with the LM@COB17 group consistently exhibiting the highest levels on days 7, 14, 21, and 28 post-immunization. Following day 21 post-immunization, the LM@COB17 group demonstrated considerably greater secretion of antigen-specific IgA antibodies than the COB17 group (*p* < 0.05).

Furthermore, we measured the total SIgA levels of jejunum mucus and the levels of SIgA-related cytokines on day 28 after immunization. As shown in Figures 6F,G, the SIgA levels in the intestinal mucosa of mice on the 28th day following vaccination were consistent with the aforementioned findings, with the LM@COB17 group exhibiting the highest SIgA level, significantly surpassing that of the COB17 group (*p* < 0.01). As opposed to the control group, the levels of IL-5 and TGF-*β* in the COB17 and LM@COB17 groups were elevated, with the LM@COB17 group showing the highest level, and the expression of IL-5 in the LM@COB17 group being considerably greater than that in the COB17 group (*p* < 0.01). Combined with the results described above on the increase of specific IgA levels in the LM@COB17 group, it is indicated that LM@COB17 significantly enhanced antigen-specific mucosal immunity compared to COB17.

To evaluate the alterations in immune-related cytokines in spleen cells, we assessed the mRNA expression of IL-4, IL-10, IL-1β, and IFN-*γ* (Figure 6H). The vaccine group exhibited elevated expression levels of IL-4, IL-10, IL-1β, and IFN-γ, with the LM@COB17 group maintaining the highest level. This indicated that LM@COB17 enhanced the degree of immune-related cytokine mRNA expression.

To prove that LM@COB17 and COB17 immunized mice can neutralize not only *C. perfringens* type A infection, but also *α*-toxin infection, we performed α-toxin neutralization assays using the serum of immunized mice. As shown in Supplementary Figure S1, after co-incubation of α-toxins with serum of the control group, an external hemolytic ring was visible on the platelets. However, after incubation with the serum of LM@COB17 and COB17 groups, there was no external hemolytic ring, indicating that both groups could neutralize the attack of α-toxins. Moreover, histopathological analyses found the intestinal villi of mice in the COB17 and LM@COB17 groups remained relatively intact, with the connective tissue of the lamina propria and submucosa showing only a small number of inflammatory cell infiltrates (Figure 6I), this suggested oral administration of LM@COB17 effectively mitigates damage to the intestinal barrier.

### 3.7 LM@COB17 could produce rapid immunity and cross-protection

**Figure 7:**
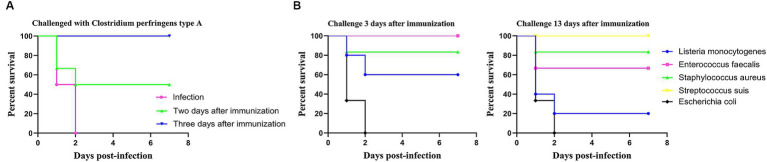
Rapid immunity and cross-protection. **(A)** Survival rate of mice challenged with *C. perfringens* type A for 2 and 3 days after immunization. **(B)** Survival rate of mice challenged with *Listeria monocytogenes*, *Enterococcus faecalis*, *Staphylococcus aureus*, *Streptococcus suis*, and *Escherichia coli* for 3 and 13 days after immunization. Each experimental group consisted of 6 mice per group.

To evaluate whether LM@COB17 can induce rapid immunity in mice, the mice were immunized and then challenged after 2 and 3 days. The survival rate of the mice was monitored from day 0 to 7 after the challenge. As illustrated in Figure 7A, it was observed that LM@COB17 provided partial protection to mice at 2 days post-immunization, but it conferred complete protection at 3 days post-immunization. These findings indicate that LM@COB17 can elicit rapid immunity in mice.

As LM@COB17 demonstrated the ability to induce rapid immunity in mice, a cross-protection test was conducted. As depicted in Figure 7B, the results revealed that oral LM@COB17 provided incomplete protection against *L. monocytogenes* and *S. aureus*, but conferred complete protection against *E. faecalis* and *S. suis*, while exhibiting no protective effect against *E. coli* after 3 days of oral immunization. Furthermore, after 13 days of oral immunization, we observed that it had a completely protective effect on *S. suis*, but had an incompletely protective effect on *E. faecalis*, *L. monocytogenes*, and *S. aureus*, with no protective effect on *E. coli*. It is evident that LM@COB17 could induce cross-protection in mice.

### 3.8 Transcriptome analysis suggested that LM@COB17 probably enhanced immunity by promoting the digestion and absorption of antigens

**Figure 8:**
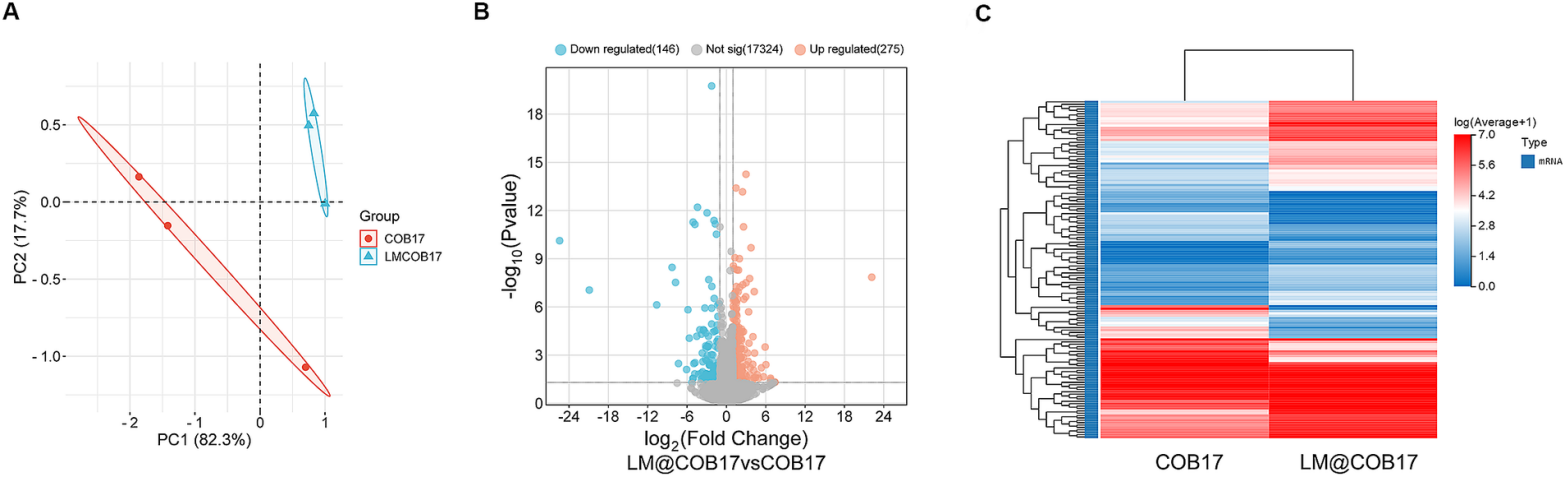
mRNA expression profile changes. **(A)** The results from the PCA analysis of the small intestine RNA-sequencing data. **(B)** The volcano plot analysis of expressed genes. **(C)** The Heatmap plot of differentially expressed genes.

To evaluate the impact of LM@COB17 on the mouse intestine, 3 days after immunization, three mice were randomly selected for sacrifice, and transcriptomic analysis of the small intestine was performed. The PCA analysis (Figure 8A) demonstrates the good separation of samples from the COB17 and LM@COB17 groups. Additionally, the Volcano plot analysis (Figure 8B) identified 275 up-regulated genes and 146 down-regulated genes in the LM@COB17 group contrasted with the COB17 group. The Heatmap plot of DEGs also illustrated the upregulation and downregulation of gene expression between the two groups (Figure 8C). The results showed that LM@COB17 caused gene expression differences compared with the COB17 group.

**Figure 9:**
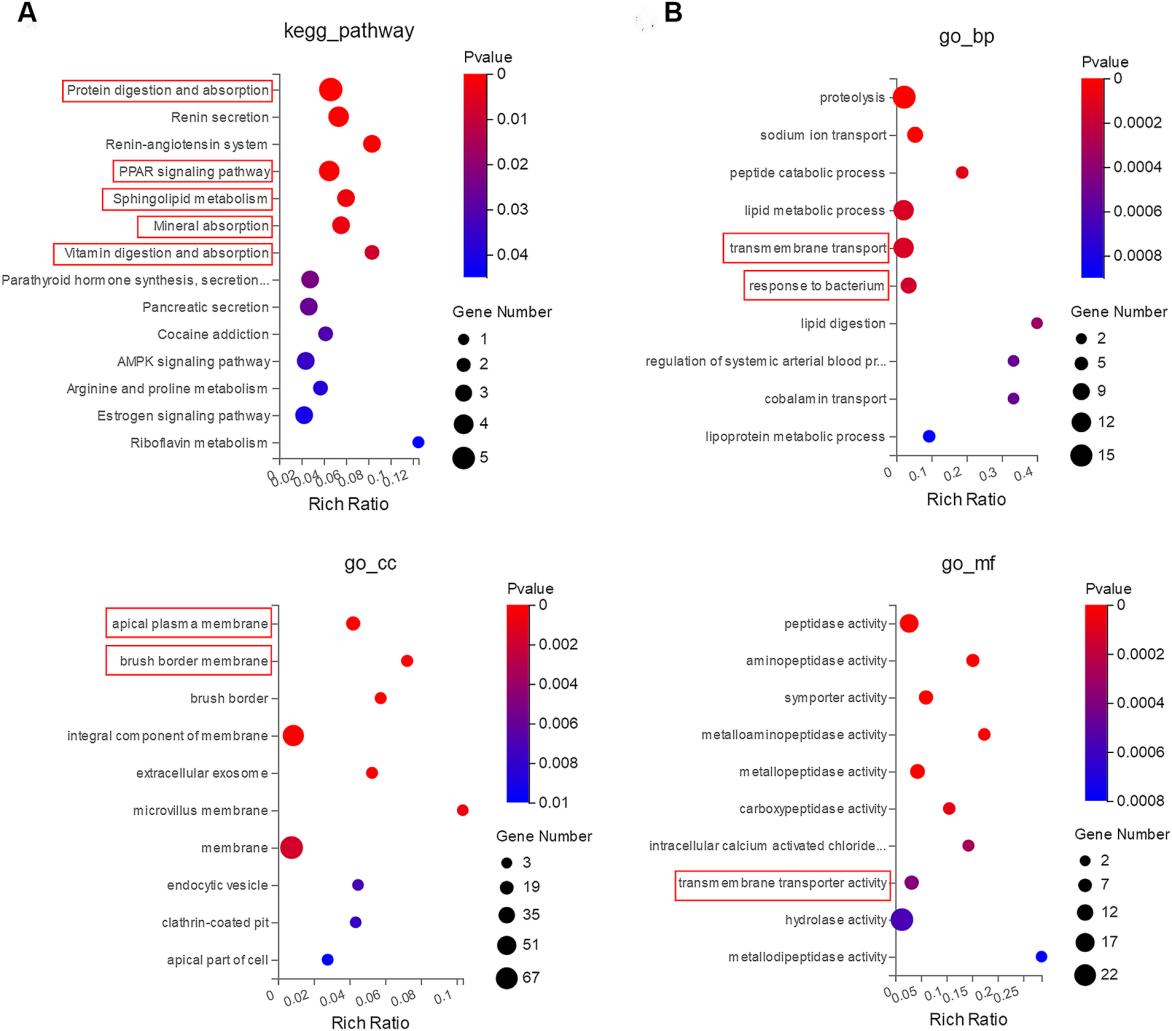
Enrichment analysis of GO and KEGG. **(A)** KEGG route enrichment statistics. **(B)** GO enrichment statistics. The *X*-axis shows the Rich Ratio, Rich Ratio = Term Candidate Gene Num/Term Gene Num. The *Y*-axis corresponds to the KEGG pathway or GO terms. The color of the dot represents the pvalue, and the dot size represents the number of DEGs mapped to the reference path.

To explore the regulatory function of LM@COB17, we performed KEGG and GO enrichment analysis. The results showed that the top KEGG pathways revealed that LM@COB17 enhanced intestinal digestion and absorption in mice, along with activation of sphingolipid metabolism and the PPAR signaling pathway (Figure 9A). Furthermore, the GO enrichment analysis of DEGs (Figure 9B) indicated enriched GO terms related to absorption and transport in the intestine, including transmembrane transport, apical plasma membrane, proteolysis, and response to bacterium, which were significantly influenced. The results indicated that LM@COB17 activated pathways associated with digestion, absorption, and transport.

**Figure 10:**
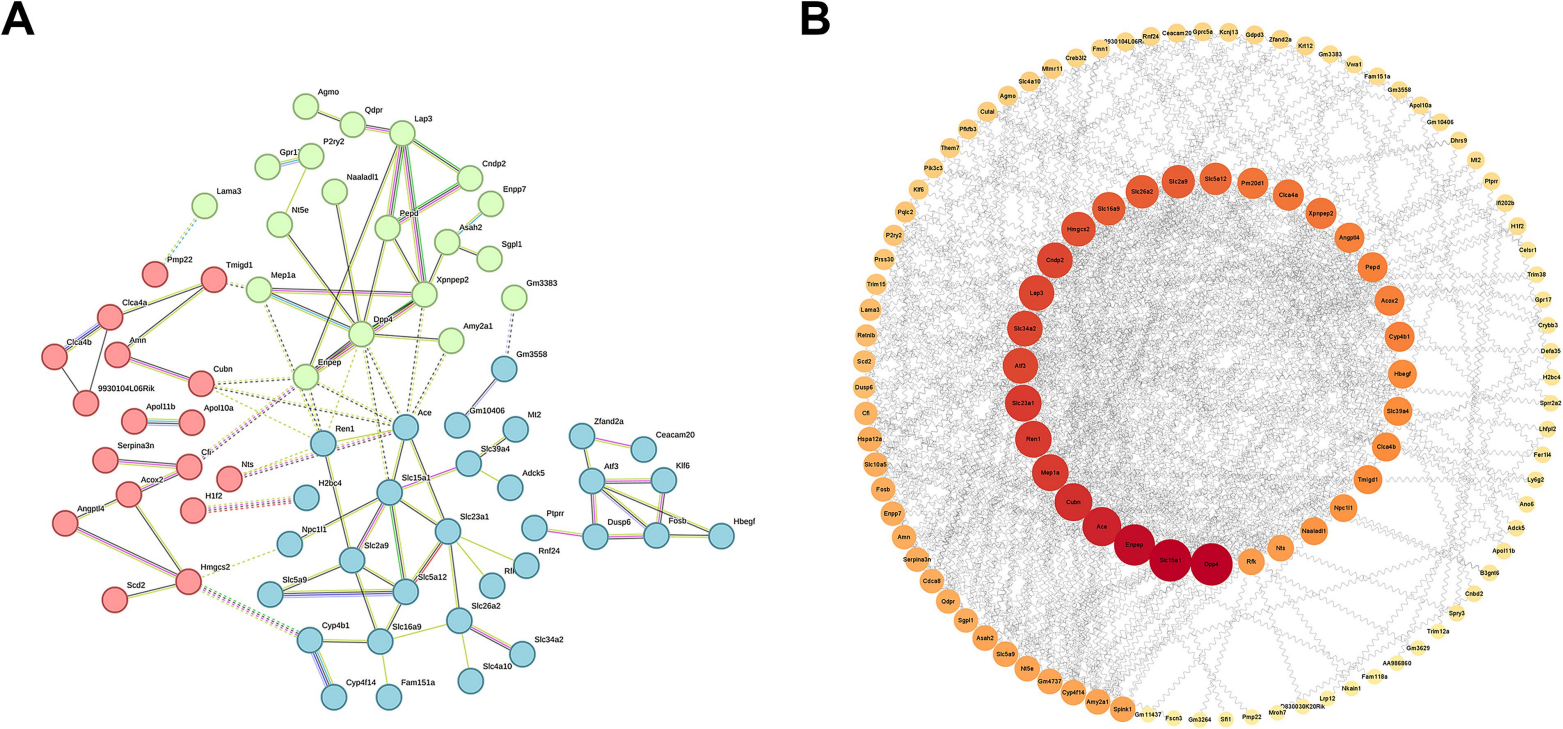
PPI network of DEGs. **(A)** Close relationships between proteins encoded by DEGs. **(B)** Identifying Hub genes from PPI networks.

To further elucidate the key genes in DEGs, we generated the PPI network through the STRING database, demonstrating the close relationship between the proteins encoded by these genes (Figure 10A). Moreover, employing the Cytoscape software, we identified Dpp4, Slc15a1, and Enpep as focal genes that act as connecting hubs (Figure 10B). Dpp4 is a helper molecule for T-cell activation. Slc15a1 belongs to the PTR2/POT transporter family, it may constitute a major route for the absorption of protein digestion end-products. Enpep is also well recognized for its ability to hydrolyze proteins. Therefore, based on the results of KEGG and GO enrichment analyses, it can be inferred that LM@COB17 potentially enhances the immune response by facilitating the uptake and translocation of antigens.

## 4 Discussion

Previous research has shown that BLPs are an effective vector for stimulating mucosal immune responses and have been utilized in the development of various vaccines (Bi et al., 2020), but currently, BLP-based vaccines are unsatisfactory for oral immunization. Additionally, artificially prepared lipid-based vesicles have been investigated for oral delivery (Bardania et al., 2017). This prompted us to wonder whether an artificially prepared lipid membrane could successfully encapsulate a BLP-based vaccine and whether the combination could enhance oral immunization with a subunit vaccine. In this study, we achieved successful preparation of a BLPs vaccine encapsulated in lipid membranes. The production process is simple, convenient, and time-efficient, with an encapsulation efficiency of 99%. The vaccine demonstrates excellent biocompatibility and stability. Furthermore, the antigens on the surface of BLP vaccines are shielded by lipid membranes, protecting them from damage in the hostile environment of the gastrointestinal tract and increasing their absorption in PPs. Our research confirmed that a single oral immunization with LM@COB17 induces up to 100% protective immunity. We also observed that LM@COB17 was able to induce rapid immunity and cross-protection in mice, raising the possibility of training immunity. Moreover, transcriptome analysis of the small intestine indicated that LM@COB17 possibly enhanced immunity by facilitating the absorption and transport of antigens in the intestines of mice.

In this study, we successfully prepared a novel oral delivery system: a display system for BLPs covered by lipid membranes. Oral administration is widely used for treating various diseases due to its cost-effectiveness, ease of administration, and safety benefits (Xu et al., 2020). However, despite these advantages, oral administration presents significant challenges, as many drugs are unstable in the gastrointestinal tract and unable to penetrate the intestinal barrier, resulting in low oral bioavailability (Bakhru et al., 2013). As the carrier of the vaccine antigen, BLPs can effectively bind the antigen and display it on their surface. These particles act as adjuvants by activating the innate immune system through interaction with toll-like receptors. To address the challenge of low bioavailability, lipid membranes were utilized to encapsulate a BLPs vaccine. The lipid membranes efficiently protected the BLP vaccine from the harsh gastrointestinal environment, enhancing its absorption in the intestines. Additionally, this approach circumvented the potential adverse reactions associated with injection immunization. Mice were conveniently and non-invasively immunized through oral delivery of COB17 enclosed in lipid membranes.

The process of preparing lipid membranes and encapsulating BLPs vaccines is highly convenient and efficient. Phospholipid molecules serve as the primary components of lipids, with soy lecithin being a major phospholipid used in lipids (Akbarzadeh et al., 2013). Furthermore, some lipid compounds, either synthetic or semisynthetic, are derived from naturally occurring precursors such as dipalmitoylphosphatidylcholine (DPPC) or dimyristoylphosphatidylcholine (DMPC) and can be utilized as lipid sources (Yingchoncharoen et al., 2016). In this study, dioleoylphosphatidic acid (DOPA) and cholesterol were dissolved in chloroform at a molar ratio of 4:1 and subsequently dried using rotary evaporation to obtain lipid membranes. These membranes were then hydrated in the vaccine solution, and the self-assembly of the lipid membranes and BLPs vaccine was achieved in less than 15 min using a vortex. This production process is characterized by its ease, reliability, environmental friendliness, and safety. Moreover, it entails a short production time and can be completed under mild conditions.

Lipid membranes exhibit a high encapsulation efficiency. In the design of lipid membrane encapsulation preparation, cholesterol was utilized to enhance the encapsulation capacity of various compounds (Kirby et al., 1980). Cholesterol naturally occurs in the cell membrane of living organisms and interacts with membrane phospholipids, playing a crucial role in eukaryotic cell functionality (Wang et al., 2017). In this study, we prepared lipid membranes with a certain ratio of DOPA mixed with cholesterol to coat our BLPs vaccine. Our findings revealed that the prepared lipid membranes exhibited extremely high encapsulation efficiency, reaching 99% fully enveloping our BLPs vaccine.

Lipid membranes and BLPs have demonstrated strong biocompatibility, making them promising materials for oral delivery. Safety and toxicological concerns are of paramount importance when evaluating the potential of these materials. The concept of lipids was first introduced by Bangham et al. (1965) and has since been established as a biocompatible and biodegradable carrier (Wang et al., 2023). Previous studies have shown that BLPs made using *Lactococcus lactis* have good biocompatibility (Bi et al., 2020). In this study, we have shown that BLPs prepared with *Lactobacillus brevis* 23,017 exhibit favorable biocompatibility. In this study, we demonstrated the safety of LM@COB17 for IPEC-J2 cells *in vitro*. After incubation with LM@COB17 for 6 and 24 h, cell viability remained unaffected. Furthermore, when compared to healthy mice, those given LM@COB17 orally did not exhibit any significant differences in body weight or histological sections of major organs. These findings indicate the satisfactory biological safety of LM@COB17 *in vivo*.

Lipid membranes can shield the antigen on the surface of BLPs vaccines from adverse gastrointestinal environments, facilitating the delivery and release of the antigen to specific sites. The hydrophilic nature of gastrointestinal peptidases, sulfhydryl compounds like glutathione, and dietary proteins prevents their entry into the lipophilic phase of the lipid membrane, thus safeguarding the vaccine against enzymatic degradation and unintended thiol/disulfide exchange reactions (Olusanya et al., 2018). In this study, TEM observation revealed that the morphology of the BLPs vaccine encapsulated by the lipid membrane was slightly damaged after treatment with simulated gastrointestinal fluid, while the morphology of the vaccine not encapsulated was severely damaged. Furthermore, flow cytometry analysis revealed that only 20% of the lipid membrane was shed after 3 h of SGF treatment, indicating relative stability of the coating membrane in SGF. Quantification of the number of particles in the solution demonstrated that after 3 h of treatment with SGF, the number of particles in the LM@COB17 solution decreased by only 26%, while COB17 decreased by 77%. As anticipated, coating with the lipid membranes could protect the integrity of COB17 in gastric acid, which was in stark contrast with uncoated COB17.

LM@COB17 elicited robust humoral and mucosal immunity in mice and a single oral immunization resulted in 100% protection. As a drug delivery system, lipid membranes have enhanced therapeutic approaches in various biomedical applications by stabilizing therapeutic compounds and overcoming barriers to cellular and tissue uptake (Ding et al., 2006). Moreover, BLPs activate the innate immune system primarily through TLR 2 and have been extensively investigated as a promising adjuvant for vaccines against a variety of infectious diseases (Keijzer et al., 2014). In this study, encapsulation of the BLPs vaccine in lipid membranes resulted in 100% protective effects after the challenge. LM@COB17 also exhibited notable advantages in delaying weight loss and reducing clinical scores in mice. Furthermore, LM@COB17 induced mice to produce higher levels of specific IgG and SIgA, as well as immune-related cytokines, suggesting that antigen was released at a specific location in the intestine, triggering robust humoral and mucosal immunity in mice. *In vitro* toxin neutralization experiments and pathological section observations also demonstrated the efficacy of LM@COB17 in preventing *C. perfringens* infection.

To our surprise, LM@COB17 induced rapid immunity and cross-protection in mice. In a previous study, mice vaccinated intranasally (IN) with the BLP-adjuvanted H1N1 A/New Caledonia-derived split virus vaccine showed complete protection against weight loss upon heterologous challenge with H1N1 A/PR/8/34 (de Haan et al., 2012). BLP is known to activate the innate immune system via TLR 2 (Ramirez et al., 2010). Prior research has demonstrated that lipid membrane can elicit innate immunity (Moghimi and Szebeni, 2003) and activate the complement system (which, upon activation, is involved in a wide range of immune and inflammatory processes) (Zahednezhad et al., 2019). Without immunological memory, the innate immune response offers an initial, generally nonspecific response to infection within hours or days. TLR 2 has been widely used to potentiate the innate immune system as an innate immunity receptor agonist (Chiang et al., 2022). Nevertheless, throughout the last 10 years, there has been evidence that innate immune cells, including macrophages, NK cells, and monocytes, can also induce metabolic and epigenetic reprogramming in cells to promote long-term memory formation. This memory is termed ‘trained immunity’ (Gu et al., 2021). Zhu et al. used zymosan (a toll-like receptor 2 agonist) to train the innate immune system of C57BL/6 mice and found that the trained immune system can protect against five different types of bacteria. The protection conferred by immunity training lasted up to 8 weeks in this mouse model (Zhu et al., 2020). Another study showed that the upregulation of TLR4 expression on alveolar macrophages which was trained by whole cells of *Acinetobacter baumannii* plays an important role in vaccination-induced rapid protection (Gu et al., 2021). Similar findings in our study, LM@COB17 exhibited an incomplete protective effect on mice 2 days after immunization. However, it conferred complete protection on mice at 3 days post-immunization, suggesting a rapid induction of immunity. Additionally, 3 and 13 days after immunization, we found that LM@COB17 was cross-protective to varying degrees against *L. monocytogenes*, *S. aureus*, *E. faecalis*, and *S. suis*. Therefore, we hypothesized that LM@COB17 may induce a trained immunity response to provide rapid immunity and cross-protection in mice.

LM@COB17 promoted the absorption and transport of antigens. It has been documented that the solubilization effect of lipids encapsulating drugs can significantly increase the intestinal transmembrane permeability of these drugs during intestinal absorption (Song et al., 2011). Research has indicated the abundant presence of folate receptors on intestinal epithelial cells, combining folate with lipids can enhance the effective uptake of drugs (Wang et al., 2016). In this investigation, firstly, frozen sections of intestinal PPs show a higher uptake of LM@COB17 than COB17. Second, as shown by transcriptome sequencing results, the KEGG enrichment pathway analysis revealed that LM@COB17 treatment promoted intestinal digestion and absorption in mice, and the significant influences on GO terms associated with transmembrane transport, apical plasma membrane, proteolysis, and response to bacterium. Subsequently, we conducted a PPI network analysis and screened Dpp4, Slc15a1, and Enpep as core genes. Enpep mainly affects protein digestion. Slc15a1 (PepT1) is a transporter apically expressed along the lumen of the gastrointestinal tract, belonging to the family of proton-coupled oligopeptide transporters (POTs), and responsible for the absorption and transport of proteins (Epling et al., 2018). In the intestine, the PepT1 protein is highly expressed in the apical membrane of enterocytes located in the mouse duodenum, jejunum, and ileum (Viennois et al., 2018). While multiple distinct amino acid transporters facilitate the transportation of amino acids, the high-capacity/low-affinity peptide transporter isoform PepT1 is responsible for the proton-coupled absorption of over 8,000 di- and tripeptides (Spanier, 2014). The increased expression of PepT1 in mice of the LM@COB17 group may be due to the ability of lipid membranes to protect the antigen from degradation by gastrointestinal fluids, leading to increased antigen absorption by intestinal PPs. Subsequently, the activation of the intestinal amino acid transport system contributes to elevated expression of PepT1. DPP4, also known as CD26, can facilitate T cell proliferation and activate NF-kappa-B in a TCR/CD3-dependent manner (Gorrell et al., 2001). The NF-kappa-B family of transcription factors regulates both innate and adaptive immune responses as well as the growth and upkeep of the cells and tissues that make up the immune system at several different stages (Hayden and Ghosh, 2011). We observed that LM@COB17 induced cross-protection and stronger antigen-specific immunoprotection in mice should be related to the activation of this pathway. Therefore, we can infer that LM@COB17 promotes antigen absorption and transport in the intestine, further activating the NF-kappa-B pathway to induce a more robust immune response.

In this study, we developed a novel bionic carrier oral delivery system with a functional coating, which effectively enhances humoral and mucosal immunity in mice. Just one oral administration of LM@COB17 produced 100% protection, along with the induction of rapid immunity and cross-protection. The observation of these effects raises questions about the underlying mechanism for rapid immunity and cross-protection, as well as the potential application of this combination for the oral delivery of other antigens. Future studies will explore these questions, with the expectation that the findings will contribute to the progress and development of oral delivery of subunit vaccines.

## 5 Conclusion

In this study, we investigated a novel bionic carrier oral delivery system with a functional coating, which involved encapsulating a BLPs vaccine in a lipid membrane using a simple self-assembly method. The preparation process of the delivery system is convenient and rapid, with an encapsulation efficiency of 99%. The new delivery system demonstrates good biosafety and effectively protects the antigen from the adverse gastrointestinal environment, as well as increasing its absorption in the intestinal PPs. Our findings indicate that the BLPs vaccine coated with a lipid membrane enhances both humoral and mucosal immunity in mice and a single oral immunization resulted in 100% protection. Our results show that LM@COB17 can induce rapid immunity and cross-protection. Transcriptome analysis of the small intestine revealed that LM@COB17 enhanced the absorption and transport of antigens in the intestines of mice, suggesting a potential immune-boosting role. In conclusion, we anticipate that the design and implementation of this oral delivery system will offer a novel approach to advancing the development of oral subunit vaccines.

## Data Availability

The raw data supporting the conclusions of this article will be made available by the authors, without undue reservation. Transcriptome data can be found here: https://www.ncbi.nlm.nih.gov/bioproject/PRJNA1163751.
